# Adult hippocampal neurogenesis occurs in the absence of *Presenilin 1* and *Presenilin 2*

**DOI:** 10.1038/s41598-018-36363-7

**Published:** 2018-12-18

**Authors:** Jagroop Dhaliwal, Timal S. Kannangara, Michael Vaculik, Yingben Xue, Keren L. Kumar, Amanda Maione, Jean-Claude Béïque, Jie Shen, Diane C. Lagace

**Affiliations:** 1 Department of Cellular and Molecular Medicine, Brain and Mind Research Institute, and Neuroscience Program, University of Ottawa, Ottawa, Ontario K1H8M5 Canada; 2 Department of Neurology, Brigham and Women’s Hospital and Program in Neuroscience, Harvard Medical School, Boston, Massachusetts 02115 USA

## Abstract

Mutations in the presenilin genes (*PS1* and *PS2*) are a major cause of familial-Alzheimer’s disease (FAD). Presenilins regulate neurogenesis in the developing brain, with loss of *PS1* inducing aberrant premature differentiation of neural progenitor cells, and additional loss of *PS2* exacerbating this effect. It is unclear, however, whether presenilins are involved in adult neurogenesis, a process that may be impaired in Alzheimer’s disease within the hippocampus. To investigate the requirement of presenilins in adult-generated dentate granule neurons, we examined adult neurogenesis in the *PS2−/−* adult brain and then employ a retroviral approach to ablate *PS1* selectively in dividing progenitor cells of the *PS2*−/− adult brain. Surprisingly, the *in vivo* ablation of both presenilins resulted in no defects in the survival and differentiation of adult-generated neurons. There was also no change in the morphology or functional properties of the retroviral-labeled presenilin-null cells, as assessed by dendritic morphology and whole-cell electrophysiology analyses. Furthermore, while FACS analysis showed that stem and progenitor cells express presenilins, inactivation of presenilins from these cells, using a NestinCreER^T2^ inducible genetic approach, demonstrated no changes in the proliferation, survival, or differentiation of adult-generated cells. Therefore, unlike their significant role in neurogenesis during embryonic development, presenilins are not required for cell-intrinsic regulation of adult hippocampal neurogenesis.

## Introduction

Mutations in the Presenilin genes (*PS1* and *PS2*) are the major cause of early onset familial-Alzheimer’s disease (FAD) through a loss-of-function mechanism^1,2^. The presenilins are essential components of the multiprotein γ-secretase complex, responsible for the proteolytic cleavage of amyloid precursor protein and Notch. *PS1* knockout mice are perinatal lethal^3^, with accompanying neurogenesis defects that include a diminished neural progenitor population and reduced Notch signaling^4–6^. While *PS2* knockout mice demonstrate a mild phenotype^7^, ablation of both *PS1* and *PS2* produces early embryonic lethality^8^, suggesting that *PS2* partially compensates for the loss of *PS1*.

While strong evidence supports that presenilins regulate embryonic neurogenesis, their role in adult neurogenesis is less clear. Adult neurogenesis is a process where neural stem cells (NSCs) and progenitor cells (NPCs) give rise to new neurons in several brain regions, including the subgranular zone (SGZ) of the hippocampus. Alzheimer’s disease (AD) has been reported to compromise adult neurogenesis, with AD-associated molecular players such as presenilins, Notch 1, β-Site Amyloid Precursor Protein Cleaving Enzyme 1 (BACE1), apolipoprotein E (ApoE), and amyloid precursor protein (APP) having either intrinsic, or non-cell autonomous effects, that modulate adult hippocampus neurogenesis^9–17^. Given the links between adult neurogenesis and cognitive functions, these findings have also raised the unsolved question about whether the reduction in adult neurogenesis contributes mechanistically to exacerbate neuronal vulnerability and promote cognitive decline^13,17^.

As presenilins regulate embryogenesis and are causally linked to FAD, presenilins have been proposed to act as key contributors to AD-associated dysfunction of adult neurogenesis that could contribute to cognitive decline. Specifically, a knockdown of PS1 in NPCs was reported to be associated with impaired cognitive function^18,19^; however, there have been no studies testing the requirement of PS1 and PS2 in neural stem cells of the adult brain, nor the possible compensatory contribution of PS2^8^ in the context of adult neurogenesis. Here, we examine whether presenilins are required for neurogenesis in the adult brain. First, we examined adult neurogenesis in *PS2* knockout (*PS2*^−/−^) mice. We then investigated whether the loss of both *PS1* and *PS2* alters adult neurogenesis, with two loss-of-function models: within a PS2^−/−^ mouse line, we used a retroviral approach to ablate *PS1* selectively in dividing NPCs, and a genetic approach to inactivate inducibly *PS1* in adult NSCs and their progeny using *NestinCreER*^*T2*^ mice. Our findings show that NSCs and NPCs can proliferate and differentiate into mature and functional granule neurons of the hippocampus in the absence of presenilins. Collectively, our data provide strong evidence that presenilins are not essential for the cell autonomous regulation of adult hippocampal neurogenesis.

## Results

### Adult Hippocampal Neurogenesis is Unaltered in *Presenilin-2* Germline Knockout Mice

**Figure 1:**
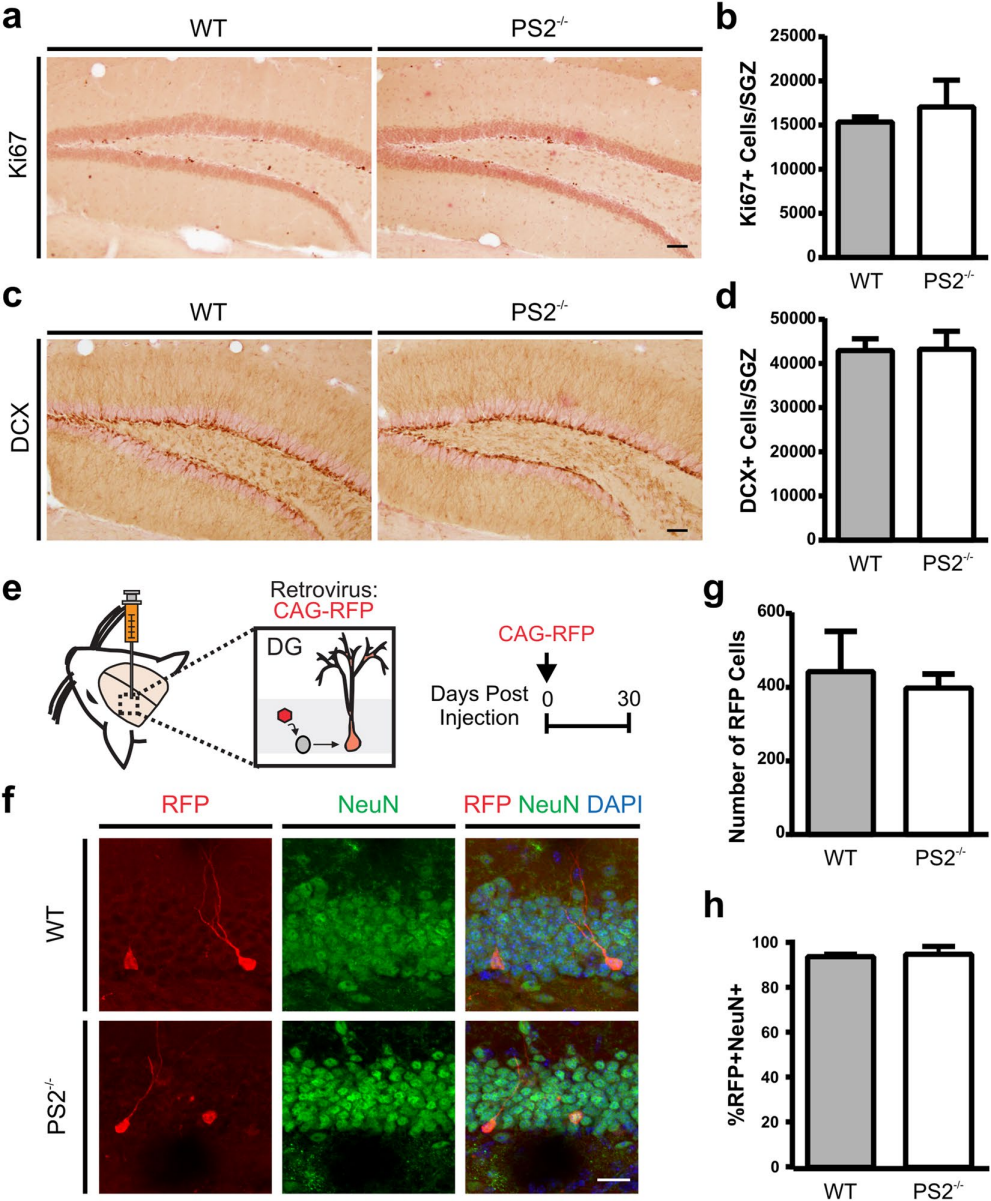
Deletion of *presenilin 2* does not affect hippocampal adult neurogenesis. (**a**) Representative images of Ki67+ dividing NPCs in wild-type (WT) and germline *presenilin 2* knockout mice (PS2^−/−^) (**b**) Quantification of Ki67+ cells shows no difference between the genotypic groups. (**c**) Representative images of DCX+ immature neurons in WT and PS2^−/−^ mice. (**d**) Quantification of DCX+ cells shows no difference between WT and PS2^−/−^ mice (n = 8 mice/genotype). (**e**) Schematic of retroviral injection into the dentate gyrus (DG) of WT and PS2^−/−^ mice. (**f **) Representative images of RFP+ cells expressing NeuN+ at 30 dpi. Scale bar, 20 μm. (**g**) Quantification of the number of RFP+ cells shows no difference between genotype. (**h**) Quantification of the proportion of RFP+ cells that express NeuN shows no difference between genotype (n = 4 mice/genotype). Scale bar, 60 μm (a,c), 20 μm (**f**). Data are presented as the mean ± SEM.

Germline *PS2* knockout (PS2^-/-^) mice are viable, therefore we assessed adult neurogenesis in the hippocampus of PS2^-/-^ mice. Quantification of the number of dividing progenitor cells, as assessed by cells expressing Ki67, revealed no differences between wild-type (WT) and PS2^−/−^ mice (Fig. 1a,b). Similarly, quantification of the number of immature neurons, assessed by expression of Doublecortin (DCX), was comparable between WT versus PS2^−/−^ mice (Fig. 1c,d).

In order to assess the survival and fate of the dividing progenitor cells, we performed bilateral injections of an RFP-tagged retrovirus into the hippocampus of WT and PS2^−/−^ mice to birthmark and track the development of the adult-generated neurons. Analysis at 30 days post infection (dpi) showed a similar number of surviving RFP+ cells within the dentate (Fig. 1e,f). Further analysis of the percentage of RFP+ cells that co-expressed the mature neuronal marker NeuN also showed no differences, with almost all cells expressing NeuN **(**Fig. 1g,h**)**. These results support previous work during embryonic neurogenesis^8^, and suggests that *PS2* is not essential for adult hippocampal neurogenesis.

### NPC Survival is Unaltered in the Absence of Presenilin1 and Presenilin2

**Figure 2:**
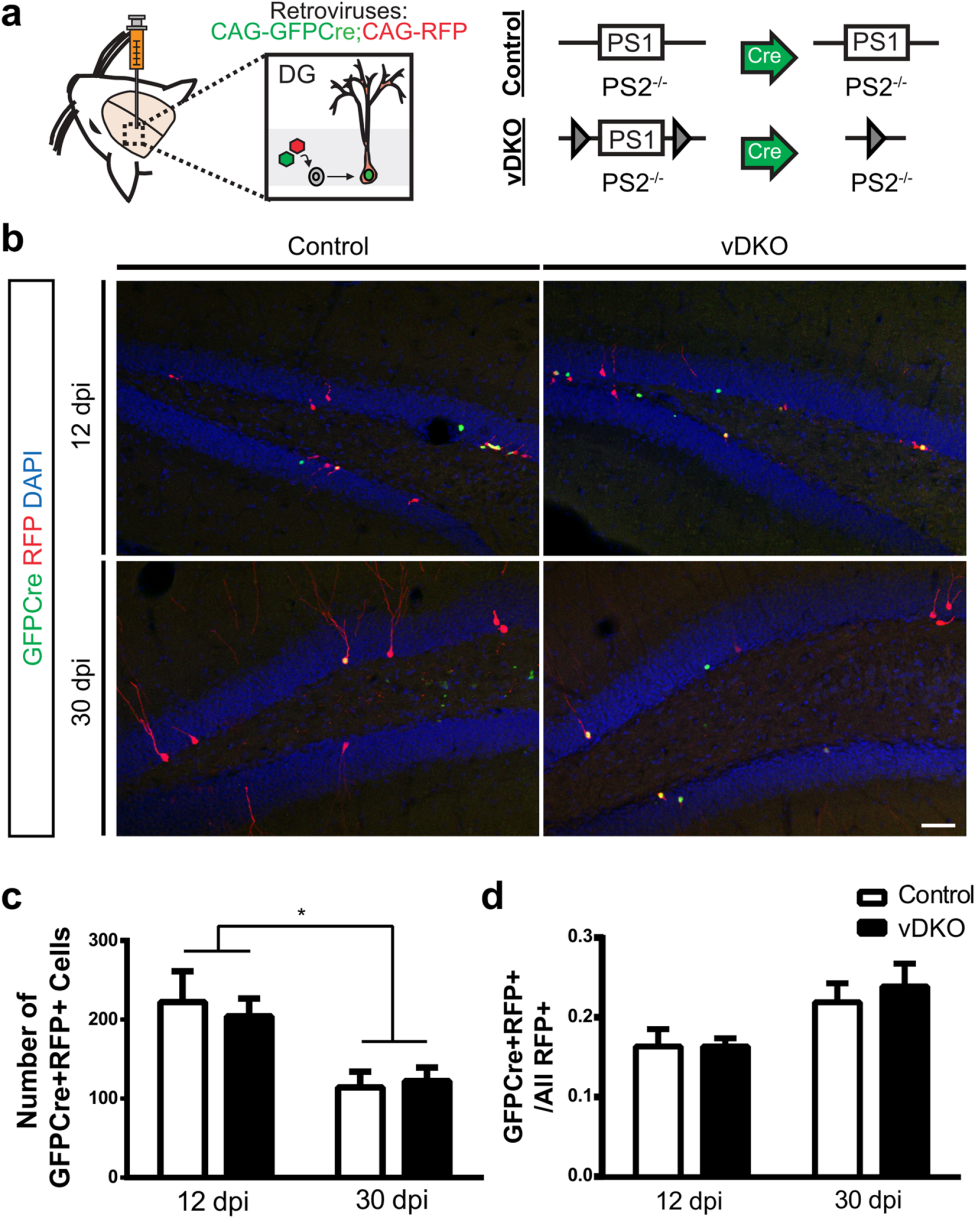
Retroviral ablation of presenilins does not affect NPC survival. (**a**) Schematic of dual retroviral (RV) approach. Two retroviruses (CAG-GFPCre, CAG-RFP) were bilaterally injected into the dentate gyrus (DG) of control (PS1^WT^;PS2^−/−^) and vDKO (viral double knockout; PS1^fl/fl^;PS2^−/−^). (**b**) Representative images of virally labeled cells showing GFPCre+ (green), RFP+ (red) and GFPCre+ RFP+ (yellow) double labeled NPCs at 12 and 30 days post infection (dpi). (**c**) Quantification of the number of GFPCre+ RFP+ virally labeled cells shows both control and vDKO cells decreasing over time (F(1, 20) = 15.9, p = 0.0007). (**d**) Quantification of survival shows no change in survival ratio ((GFPCre+ RFP+)/total RFP+) at either 12 or 30 dpi. Scale bar, 50 μm. n = 3–6 mice/genotype at 12 dpi, n = 7–8 mice/genotype at 30 dpi. Data are presented as the mean ± SEM.

*PS1* and *PS2* have overlapping functions in the developing and adult brain^20^, thus to evaluate the role of both *PS1* and *PS2* in adult neurogenesis, we fate mapped the adult dividing NPCs following a conditional ablation of *PS1* using the Cre/loxP system in PS2^−/−^ mice. Specifically, a 1:1 mixture of retroviral GFP-Cre and control RFP was bilaterally injected into PS1^fl/fl^;PS2^−/−^ (viral double knockout; vDKO) and PS1^WT^;PS2^−/−^ littermate (control) mice (Fig. 2a). At 12 and 30 dpi, vDKO and control mice had a time-dependent decrease in the number of virally-labeled cells (Fig. 2b,c). This reduction was expected since a majority of NPCs die during their development, as has been previously observed in retroviral-infected adult NPCs^21,22^. To control for this reduction in survival, we quantified the survival ratio, expressed as the fraction of double transduced (GFPCre+ RFP+) cells to total RFP+ cells, which demonstrated no change in cell survival in vDKO compared to control mice (Fig. 2d).

### NPCs Differentiate into Functional Neurons in the Absence of Presenilins

**Figure 3:**
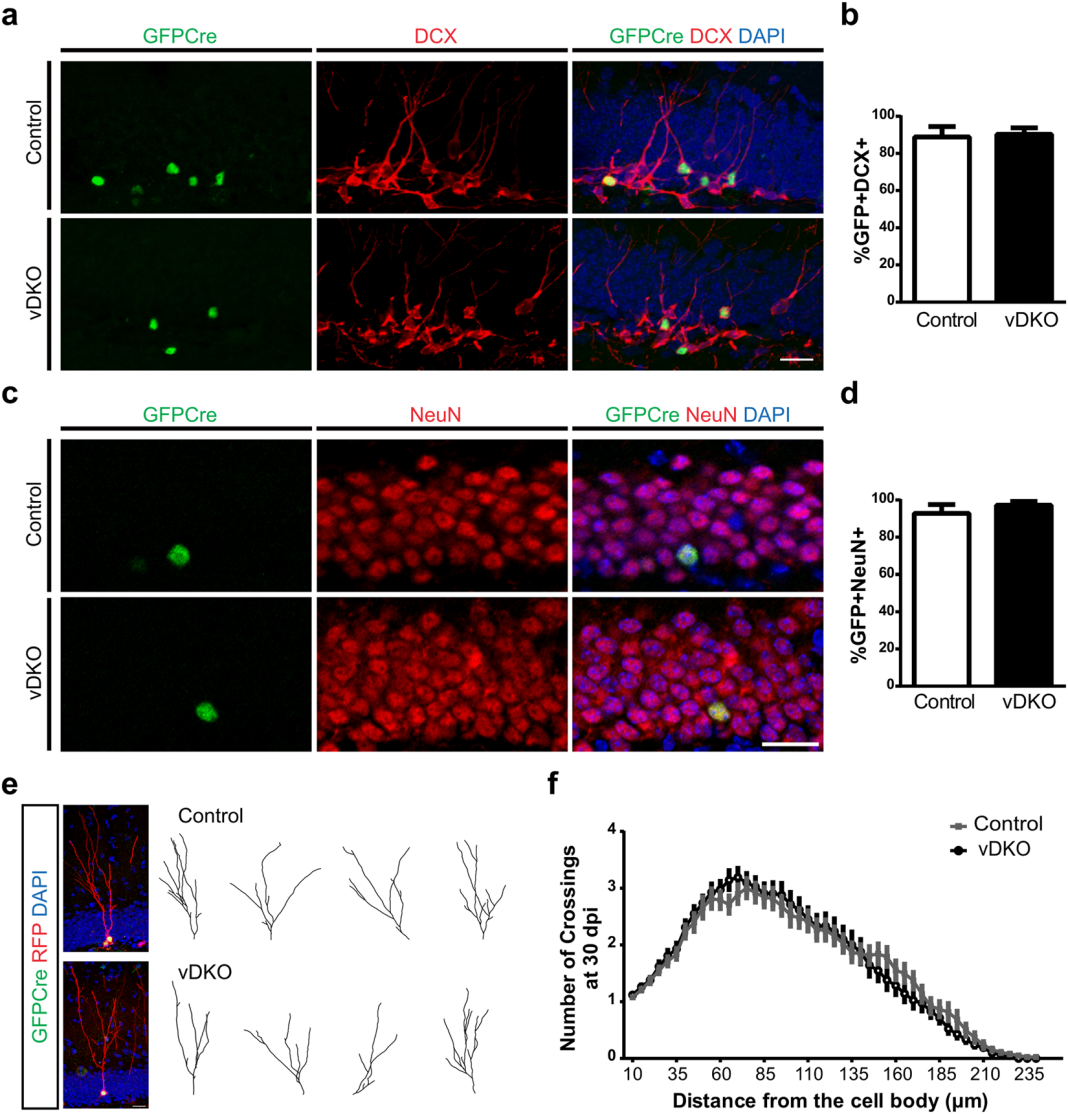
Virally labeled NPCs can differentiate into mature granule neurons in the absence of presenilins. **(a**) Representative images of GFPCre+ cells expressing DCX at 12 dpi. Scale bar, 20 μm. (**b**) Quantification of the proportion of GFPCre+ cells expressing DCX+ shows no difference between control and vDKO mice at 12 dpi (n = 2–3 mice/genotype). (**c**) Representative images of GFPCre+ cells expressing NeuN+ at 30 dpi. Scale bar, 20 μm. (**d**) Quantification of the proportion of GFPCre+ cells expressing NeuN shows that nearly all GFPCre+ cells develop into mature granule neurons irrespective of genotype (n = 3–4 mice/genotype). (**e**) Sample projections of Z-series stacks (left) and dendritic traces (right) illustrating the dendritic complexity of GFPCre+ RFP+ neurons in control and vDKO cells at 30 dpi. Scale bar, 20 μm. (**f**) Sholl analysis of dendritic complexity shows no difference between control (grey) and vDKO cells lacking presenilins (black). (n = 4 mice/genotype). Data are presented as the mean ± SEM.

To determine if the absence of presenilins altered cell fate, we quantified the proportion of GFPCre+ infected cells expressing DCX and NeuN at 12 and 30 dpi, respectively. In both control and vDKO groups, over 80% of GFPCre+ cells expressed DCX at 12 dpi, (Fig. 3a,b), and over 80% of GFPCre+ cells expressed NeuN at 30 dpi (Fig. 3c,d). In addition, quantification of dendritic morphology and the peak intersections morphological assessment of the GFPCre+ RFP+ neurons from both experimental groups at 30 dpi revealed no difference between control and cDKO cells, with values consistent to virally-labeled WT cells as we and others have previously reported^23–25^ (Fig. 3e,f). These findings suggest in the absence of presenilins, the large majority of NPCs in the DG differentiate and have a neural fate.

**Figure 4:**
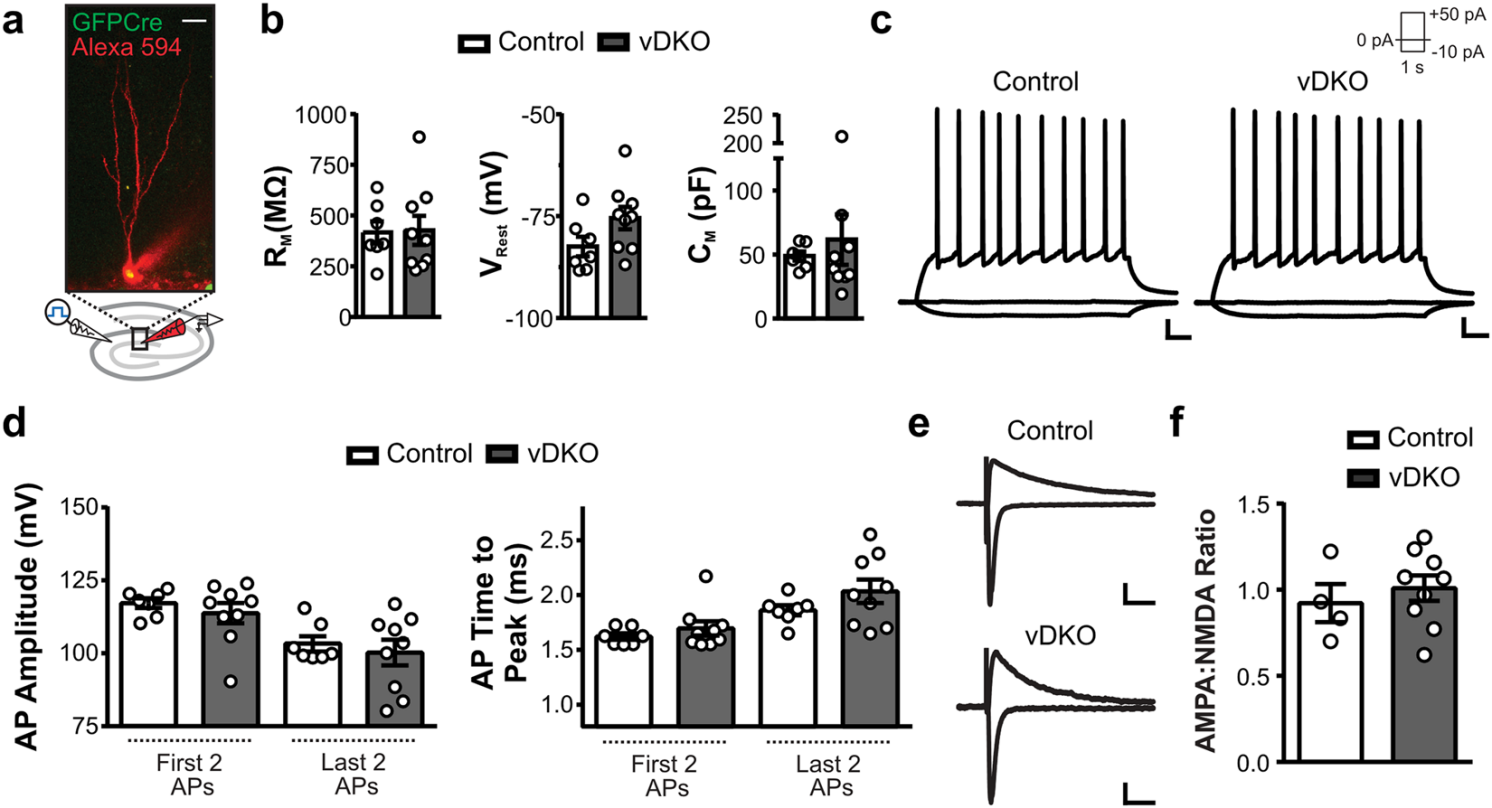
Intact electrophysiological properties of *PS*-null NPCs. (**a**) Representative two-photon image of a GFPCre+ cell filled with Alexa 594 dye. Scale bar, 10 μm. (**b**) Membrane Resistance (R_M_), resting membrane potential (V_Rest_), and estimated cell capacitance (C_M_) were similar between the two genotypes (n = 3 mice/genotype). (**c**) Representative voltage traces of action potential trains in response to direct current injection. Scale bar, 10 mV; 100 ms. (**d**) Action potential amplitude and the time to peak were comparable between the two genotypes (n = 3 mice/genotype). (**e**) Representative current traces of glutamate receptor mediated currents held at −70 and +40 mV. Scale bar, 40 pA, 50 ms (top); 20 pA, 50 ms (bottom). (**f**) AMPAR:NMDAR ratios were similar between control and vDKO in seven-eight week old virus labeled cells (n = 3–4 mice/genotype). Data are presented as the mean ± SEM.

Adult-generated cells in the DG follow a stereotypical progression towards functional integration into the hippocampal network; this involves well-characterized time-dependent alterations in intrinsic membrane properties and afferent synaptic connectivity^26,27^. To determine if presenilin ablation impacts the functional properties of maturing NPCs, we performed whole-cell electrophysiology at 6–8 weeks post retroviral labeling (Fig. 4a). The passive membrane properties were similar between cells from control and vDKO mice, with values consistent with GFP+ cells from wild-type mice (**see** Supplemental Table S1), as well as naïve virally-labeled adult-generated granule cells in previous reports^26,27^ (Fig. 4b). The ability of cells from vDKO mice to fire trains of action potentials in response to direct current injection was indistinguishable to that observed in controls (Fig. 4c,d). To examine afferent synaptic connectivity, we recorded glutamatergic excitatory postsynaptic currents (EPSCs) at the medial perforant path-granule cell synapse. Despite previous reports suggesting that presenilins modulate the function of NMDA receptors in the CA3-CA1 regions^28^, we did not detect any significant differences in the AMPA/NMDA ratio of EPSCs recorded from control and vDKO mice (Fig. 4e,f). Together, these results indicate that NPCs can develop into functional neurons in the adult DG in the absence of presenilins.

### Presenilins are not Required for Running-Induced Neurogenesis

**Figure 5:**
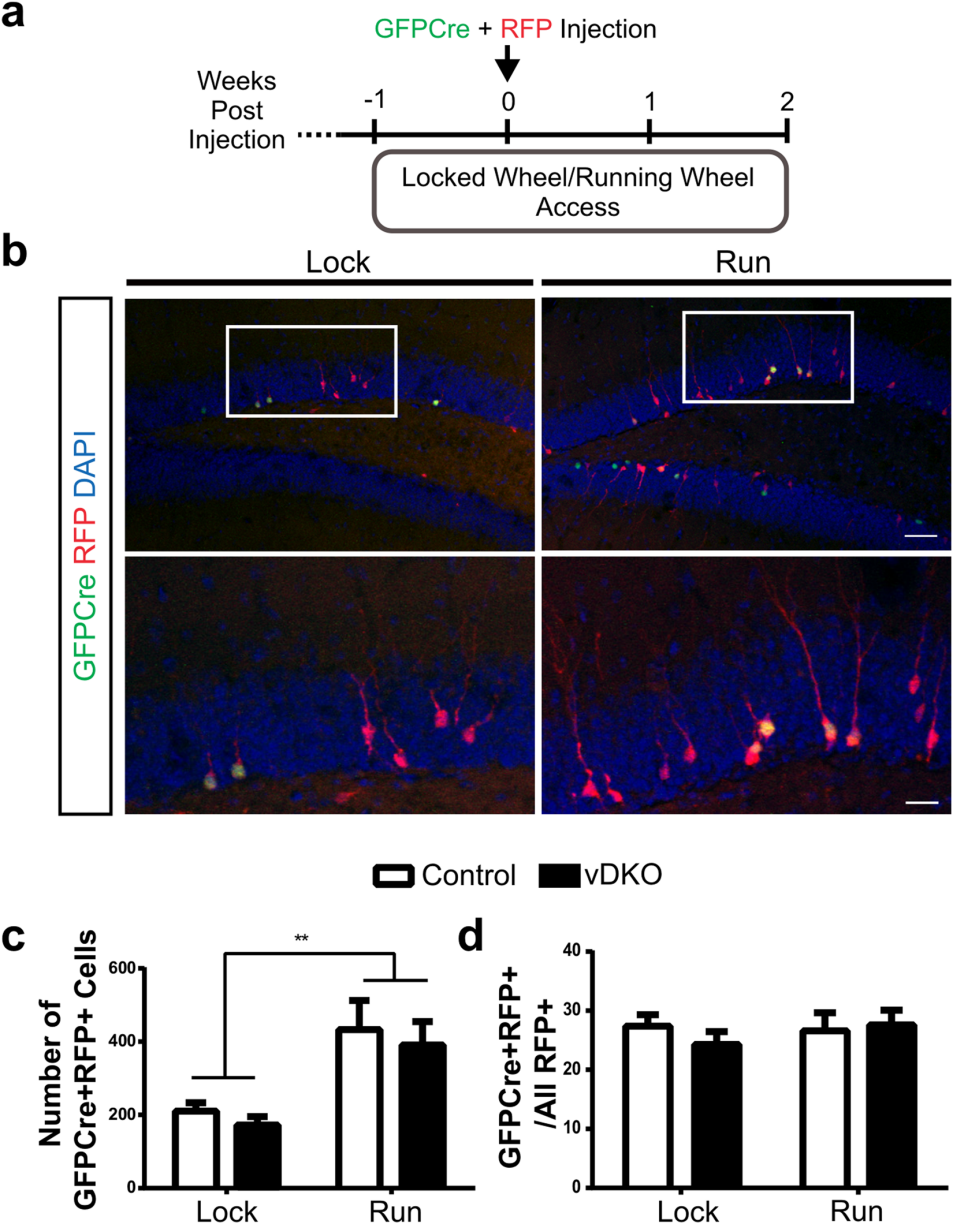
Running-induced neurogenesis occurs in the absence of presenilins. (**a**) Timeline of experiment. (**b**) Representative images of virally labeled cells in the dentate gyrus of mice with locked, non-functional wheel (lock) and functional running wheels (run). Scale bar, 50 μm (upper), 20 μm (lower). (**c**) Quantification of number of GFPCre+ RFP+ virally labeled cells significantly increased with running in both control and vDKO groups (F(1,28) = 20.7, p < 0.0001). (**d**) Quantification of survival shows no change in survival ratio following running in control and vDKO mice. (n = 9 mice/genotype for lock wheels; 7 mice/genotype for running wheels). Data are presented as the mean ± SEM.

Increasing adult neurogenesis via voluntary exercise has previously been shown to unmask an extrinsic role for various *PS1* mutations in adult hippocampal neurogenesis^29,30^. Thus, to determine if *PS1* has a cell-intrinsic function when neurogenesis is enhanced, the survival of control and vDKO cells was assessed following three weeks access to either a functional or non-functional (locked) running wheel (Fig. 5a). Both control and vDKO mice that had access to functional running wheels ran similar distances (10.5 ± 3.2 km vs. 9.4 ± 1.6 km) over the three-week period. As expected, running significantly increased the number of virally labeled GFPCre+ RFP+ cells (Fig. 5b,c). There was, however, no difference in the number of surviving cells in either the vDKO and control mice, which resulted in similar survival ratio between the two genotypes (Fig. 5d). Together, these results suggest that presenilins are not intrinsically required for running-induced neurogenesis.

### Presenilins are not Essential for the Development of Adult NSCs and their Progeny

**Figure 6:**
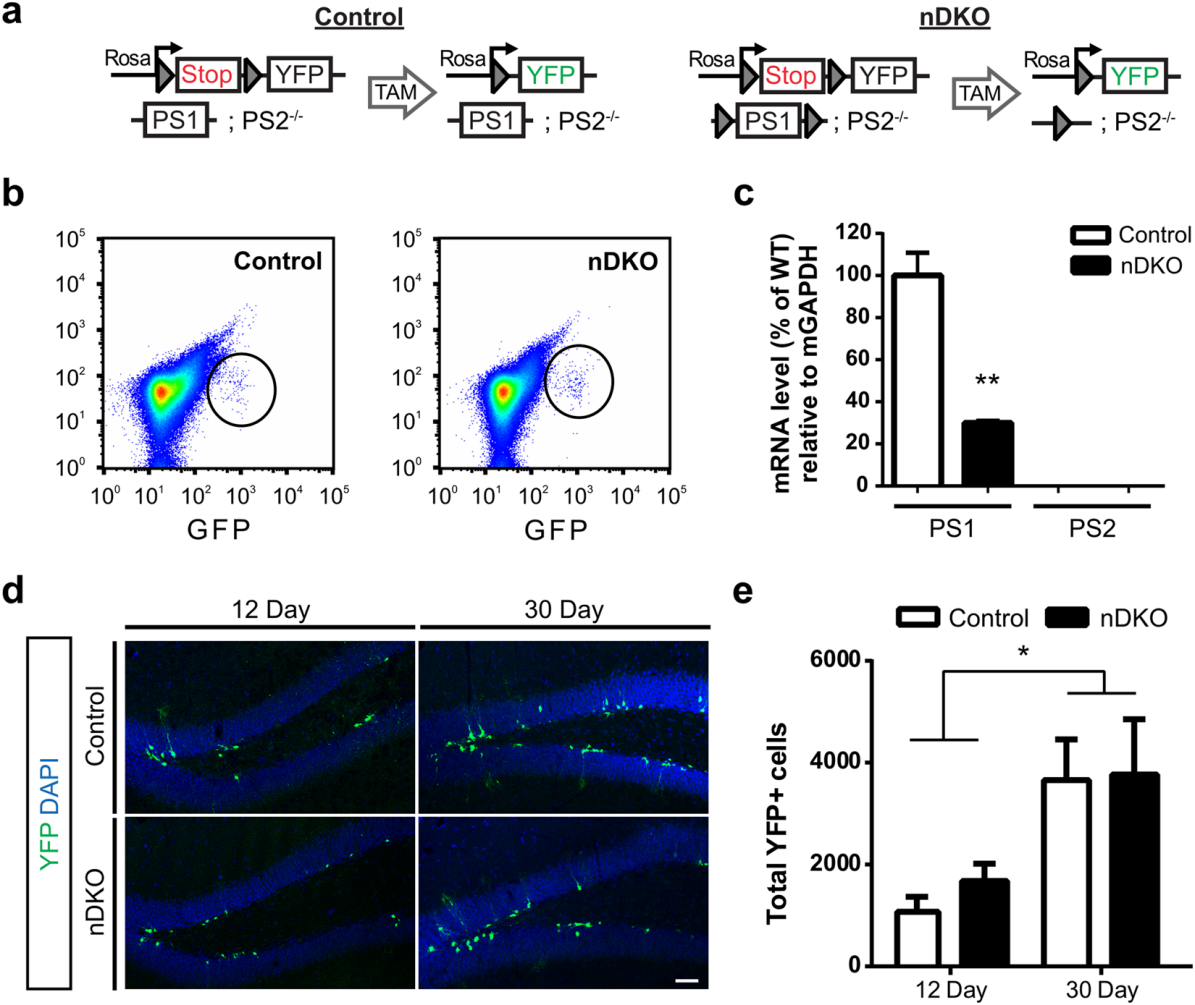
Ablating presenilins using nestin-driven inducible transgenic mouse (nDKO) does not alter the number of stem and progenitor cells and their progeny. (**a**) Schematic of recombination induced by tamoxifen (TAM) in control (NestinCreER^T2^;R26RYFP; PS1^WT^;PS2^−/−^) and nDKO (nestin-driven double knockout; NestinCreER^T2^;R26RYFP; PS1^fl/fl^;PS2^−/−^) mice. (**b**) Density-scatter plots of FACS-isolated cells from the dentate gyrus of control (left) and nDKO mice (right). (**c**) qPCR on YFP+ sorted cells show reduced PS1 mRNA in nDKO cells compared to control cells (t(4) = 6.444, p < 0.005), and no PS2 mRNA detected in either cell group. (**d**) Representative images of YFP+ cells at 12 and 30 days post-TAM in control and nDKO mice. Scale bar, 50 μm. (**e**) Quantification of the total number of YFP+ cells shows an accumulation of recombined cells at 30 days compared to 12 days post-TAM (F(1,39) = 11.2, p < 0.005), with no differences between the genotypes. (n = 9–13 mice/genotype at 12 day, 10–11 mice/genotype at 30 day). Data are presented as the mean ± SEM.

In the developing brain, presenilins are essential for stem cell maintenance, as embryonic presenilin ablation leads to progenitor cell pool depletion and premature neuronal differentiation^4^. To identify if PS1 and PS2 were present in adult NSC/NPCs, we first analyzed FACS-isolated GFP+ cells from the adult DG of Nestin-GFP reporter mice^31^ and found that GFP+ cells expressed mRNA for both *PS1* and *PS2* (**see** Supplementary Fig. S1), consistent with previous reports^32,33^. To determine the role of presenilins in adult NSCs, we generated NestinCreER^T2^;R26R-YFP;PS1^fl/fl^;PS2^−/−^ (nestin-driven double knockout, nDKO) and NestinCreER^T2^;R26R-YFP;PS1^WT^;PS2^−/−^ (control) mice. In these mice, tamoxifen (TAM) administration induces expression of YFP in control and nDKO mice, as well as specific ablation of PS1 in nDKO mice (Fig. 6a). Analysis of FACS-isolated YFP+ cells from control and nDKO mice identified that YFP+ nDKO cells had a significant reduction in PS1 mRNA in comparison to control cells (Fig. 6b,c). As expected, there was no expression of PS2 mRNA in YFP+ cells from nDKO or control mice (Fig. 6b,c). Examination of YFP+ cells in the nDKO mice showed an accumulation between 12 and 30 days post TAM injection, which is expected in this model since recombination occurs in the stem and progenitor cells, as well as their progeny (Fig. 6d,e). Notably, there was no difference between the number of YFP+ cells in control and nDKO mice, at either 12 or 30 days post TAM. Additionally, quantification of the number of YFP+ cells at 30 days post TAM in WT mice (NestinCreER^T2^;R26R-YFP;PS1^WT^;PS2^WT^; YFP+ cells = 4548 ± 785, n = 9), revealed no significant difference from the control or nDKO mice (One-Way ANOVA, F = 0.27, ns). Together these data suggesting that presenilin ablation from NSCs does not alter cell production.

**Figure 7:**
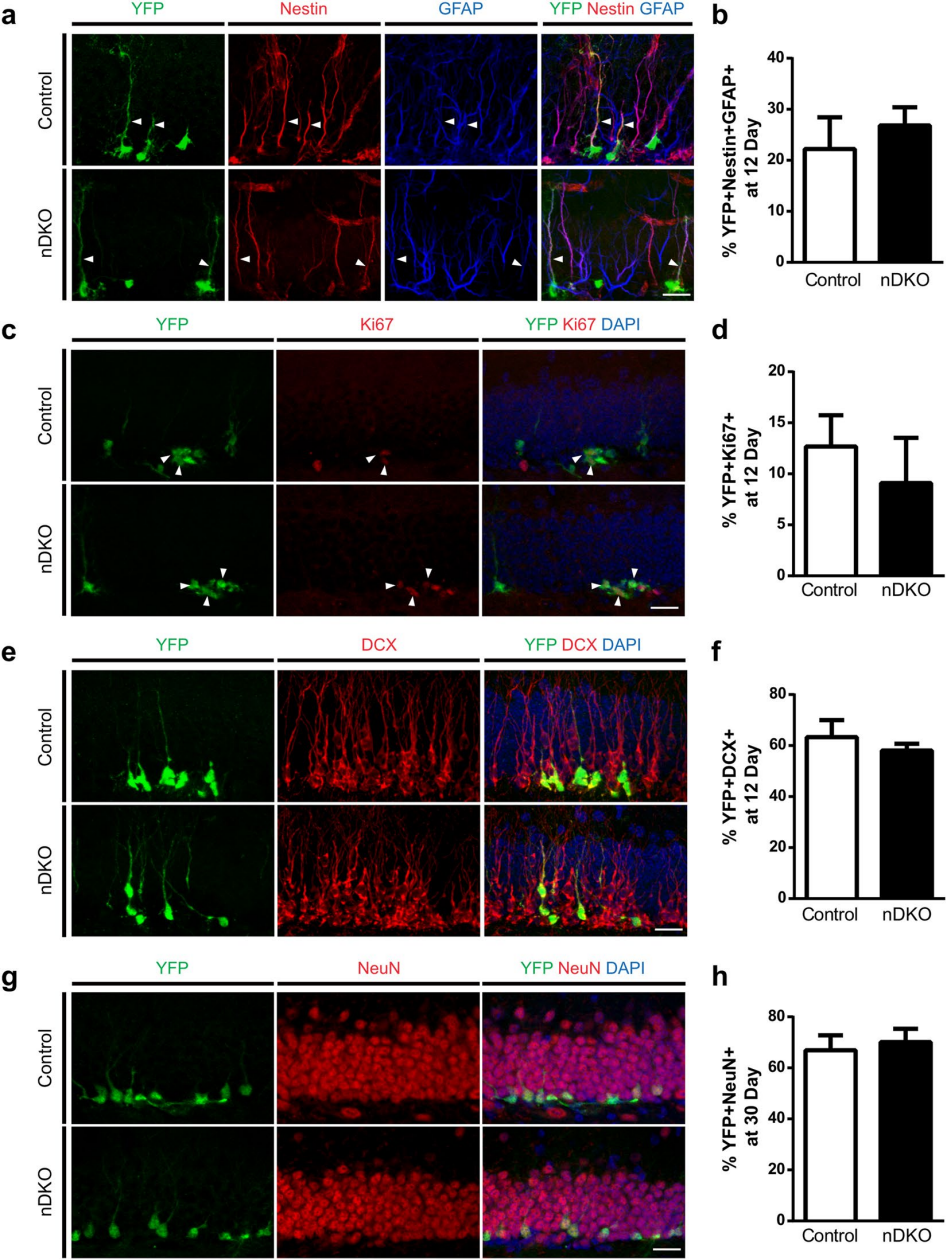
Ablating presenilins from nestin-expressing and progeny does not affect neurogenesis. (**a**) Representative images of YFP+ cells expressing the NSC markers nestin and GFAP (arrowheads) at 12 days post tamoxifen (TAM). (**b**) Quantification of proportion of YFP+ Nestin+ GFAP+ cells among all YFP+ population shows no change between genotypes (n = 4 mice/genotype). (**c**) Representative images of YFP+ cells expressing cell division marker Ki67 (arrowheads) at 12 days post TAM. (**d**) Quantification of proportion of YFP+ Ki67+ cells among all YFP+ population shows no change between genotypes (n = 6 mice/genotype). (**e**) Representative images of YFP+ cells expressing immature neuron marker DCX at 12 days post TAM. (**f)** Quantification of proportion of YFP+ DCX+ cells among all YFP+ population shows no change between genotypes (n = 6 mice/genotype). (**g)** Representative images of YFP+ cells expressing post-mitotic neuronal marker NeuN at 30 days post TAM. (**h**) Quantification of proportion of YFP+ NeuN+ cells among all YFP+ population shows no change between genotypes (n = 3 mice/genotype. Scale bars (a,c,e,g), 20 μm. Data are presented as the mean ± SEM.

To determine if removing presenilins may alter the proportion of NSC population, the recombined (YFP+) cells were phenotyped using two markers found in the radial processes of NSCs: glial fibrillary acidic protein (GFAP) and nestin (Fig. 7a, arrowheads). Both control and nDKO mice had a similar proportion of recombined radial glia-like NSCs at 12 days post TAM (Fig. 7b). Additionally, there were no differences between groups in the proportion of proliferating (Ki67+) cells (Fig. 7c,d) or immature (DCX+) neurons at 12 days post TAM (Fig. 7e,f). The nDKO and control mice also had a similar proportion of recombined neurons that expressed both DCX and NeuN at 30 days post TAM (Fig. 7g,h). Together, these results show that, unlike in the developing brain, presenilins do not influence cell intrinsic regulation of NSCs to modulate adult neurogenesis. In combination with our retroviral findings that targeted removal of presenilins from dividing NPCs, these findings also strongly suggest that presenilins are not required for the development of adult-generated granule neurons in the DG.

## Discussion

In this study, we examine whether presenilins, a leading contributor to FAD, have a cell autonomous role in hippocampal adult neurogenesis by examining PS2^−/−^ mice and by ablating *PS1* and *PS2* in NSCs and NPCs using two independent models. In the absence of presenilins, NSCs and their progeny demonstrated no significant alterations in stem cell maintenance, proliferation, or differentiation, and showed electrophysiological properties similar to that of naïve adult-generated granule neurons. Additionally, removal of presenilins did not affect adult neurogenesis induced by running. Thus, we conclude that the presenilins are not cell intrinsic mediators of adult hippocampal neurogenesis.

It is perhaps not surprising that PS2^−/−^ mice did not have an altered adult neurogenic phenotype given that *PS2* ablation does not alter the processing of APP^34^ or neurogenesis in the developing brain^8^. However, *PS2* may compensate for *PS1* during embryogenesis^8^, necessitating a concurrent ablation of *PS1* and *PS2* to understand the role of presenilin in adult neurogenesis. Surprisingly, our findings suggest *PS1* and *PS2* are dispensable for adult neurogenesis. Thus, presenilins can be added to the growing list of regulators of neurogenesis that have differential roles within the context of embryonic versus adult neurogenesis^35,36^.

The lack of a cell-intrinsic role for *PS1* on adult neurogenesis was unexpected given the striking role for *PS1* in the developing brain. Indeed, embryonic ablation of *PS1* leads to a significant depletion of the NSC/NPC pool due to the early exit from cell cycle, and premature differentiation into neurons^4^ which is attributed to a blockade of Notch signaling^5^. Notch is a substrate of γ-secretase, of which presenilin is the catalytic subunit, and is required for maintenance of embryonic neural stem cells^37^. Functional analysis of Notch1 and the Notch-pathway genes in adult NSCs have revealed ablation of Notch1 or its downstream transcriptional effectors such as RBPJκ_,_ depletes the NSC pool and suppress hippocampal neurogenesis, similar to its effects in the embryo^12,38,39^. Furthermore, conditional inactivation of Notch1/2 in postmitotic excitatory neurons of the postnatal forebrain didn’t result in similar phenotypes as conditional inactivation of presenilins using the same *CaMKIIα-Cre* transgenic mouse^40^. As we found that the adult NSC population was not modified by presenilin ablation, Notch signaling may occur independently of presenilins within the adult NSCs, thus future work is required to define the relationship between Notch and presenilins in the adult NSCs, as well as postmitotic neurons of the adult cerebral cortex.

Given that no change in adult neurogenesis was observed when presenilins were removed, our findings also suggest that the actions of presenilins within the neurogenic cells are unlikely to mediate the cognitive decline observed in FAD. Thus our studies add to the list of preclinical studies (reviewed by others^14–16,41^) that highlight the variability in the adult neurogenesis phenotypes in different presenilin and the amyloidogenic mouse models of AD. These differences also contribute to the debate about the causal versus consequential role of adult neurogenesis and AD-associated cognitive decline. In support of a causal role for neurogenesis in cognitive function, a recent study has suggested that a complete ablation of neurogenesis in young, pre-symptomatic FAD-linked APPswe/PS1ΔE9 mice can produce cognitive deficits and enhance tau hyperphosphorylation^42^. Interestingly, using this same FAD mouse model, a gene-targeting strategy to enhance the neuronal fate, maturation and synaptic integration of adult-generated neurons was reported to be able to rescue hippocampal memory deficits^43^. These results suggest that targeting the adult-generated cells may be viable and sufficient to restore cognitive decline as a regenerative medicine approach. The implications of our findings, however suggest that it is unlikely the absence of presenilins alone within adult-generated cells is a strong contributor to FAD-associated deficits.

Our findings, which specifically address the cell-intrinsic role of presenilins using retrovirus and targeted inducible transgenic approaches, do not preclude the possibility of a non-cell autonomous role for presenilins in modulating neurogenesis and cognitive function. Notably, there is growing evidence for adult neurogenesis to be regulated by a non-cell autonomous effect due to *PS1* expression in cells surrounding NSCs and NPCs. For example, mutant mice with deletions of *PS1* and *PS2* from postmitotic neurons show increases in cell density in the DG that is associated with enhanced neurogenesis^44^. Also, the enrichment-induced neurogenesis deficits observed in mice overexpressing *PS1* mutants in neuronal and non-neuronal cells can be rescued if the mutant transgene is ablated from forebrain neurons, supporting a non-cell autonomous mechanism^45^. The non-cell autonomous roles of presenilins may also account for why our finding could conflict with previous studies that showed knockdown of *PS1* can reduce adult neurogenesis^18,19^. These studies use a lentiviral approach to knockdown *PS1* and differences in our results may be attributed to the capacity of lentiviruses to infect a broad population of cells including NSCs, NPCs, as well as non-adult generated mature granule neurons^18,19^. Thus the lentiviral approach may have both cell autonomous and non-cell autonomous effects. Thus, future work also remains to explore the non-cell autonomous role of presenilins in adult neurogenesis, but our work, in combination with others, strongly support that presenilins do not have a cell intrinsic function in the regulation of adult hippocampal neurogenesis.

## Methods

### Animals and tamoxifen administration

All experiments were approved by the University of Ottawa Animal Care Committee, in accordance with the Guidelines of the Canadian Council on Animal Care. This study utilized a variety of published transgenic mice including: (1) Nestin-GFP reporter mice to label nestin-expressing NSCs and NPCs^31^, (2) NestinCreER^T2^; R26R-YFP to modify nestin-expressing NSCs and NPCs^46^, (3) floxed *PS1* (PS1^fl/fl^)^47^ and (4) embryonic *PS2* knockout (PS2^−/−^)^7^. The PS1^fl/fl^ mice were bred on a PS2^−/−^ knockout background to create, for the retroviral experiments, PS1^fl/fl^;PS2^−/−^ (here after referred to as viral double knockout; vDKO) and PS1^WT^;PS2^−/−^ (control) mice. For tamoxifen inducible experiments, quadruple inducible NestinCreER^T2^;R26R-YFP;PS1^fl/fl^;PS2^−/−^ (here after referred to as nestin double knockout; nDKO) and NestinCreER^T2^;R26R-YFP;PS1^WT^;PS2^−/−^ (control) mice were created by breeding R26R-YFP;PS1^WT/fl^;PS2^−/−^ mice and NestinCreER^T2^;PS1^WT/fl^;PS2^−/−^ mice. This allowed for the creation of littermate experimental and controls that were group-housed (2–5 per cage). Both male and female mice were utilized between 6–9 weeks of age and were maintained on a 12 hour light-dark cycle with free access to food and water.

To induce CreER^T2^ mediated recombination, mice were administered tamoxifen (160 mg/kg, dissolved in 10% EtOH/90% sunflower oil) via daily intraperitoneal (*i.p*.) injections for five days, as has been shown before^46^.

### Generation and *in vivo* injection of retroviruses

Retroviral expression plasmids used to express GFPCre and/or RFP in proliferating cells were provided by Dr. Fred Gage^21^ and retroviruses were made as previously described^48^. High titers of retroviruses (4 × 10^8^ units/ml) were produced by co-transfection of the GFPCre or RFP expression plasmids, VSVG and the packaging plasmid into HEK293T cells followed by ultracentrifugation of the viral supernatant.

A 1:1 mixture of CAG-GFPCre & CAG-RFP retroviruses were bilaterally injected into the dentate gyrus (DG) (1.5 μL/injection at 0.2 μL/minute) in anaesthetized (2% Isoflurane) mice using stereotaxic surgery and coordinates of antero-posterior = −1.7, lateral = +1.2/−1.2, ventral = −2.4 of bregma. Mice were sacrificed at 12 and 30 days post-infection (dpi) for cell counts, phenotyping and dendritic analysis.

For the running experiment, mice were singly housed with free access to a low profile wireless running wheel or a locked wheel (Med Associates) for one week prior to, and for two weeks post retroviral infection prior to perfusion.

### Fluorescence-Activated Cell Sorting (FACS) Analysis and PCR

The dentate gyri were isolated from mice (5–7 weeks of age) and placed in oxygenated artificial cerebrebrospinal fluid used for FACS (FACS-aCSF), consisting of (in mM): 124 NaCl, 5 KCl, 1.3 MgCl2 · 6H2O, 2 CaCl2 · 2H2O, 26 NaHCO3, and 1X penicillin-streptomycin (10,000 U/mL; ThermoFisher) (pH = 7.4). Tissue was gently chopped using a sterile scalpel blade, spun down and incubated (ten minutes, 37 °C) in 500 uL/tube of digestion media [20 U/mL papain (Worthington Biochemicals), 12 mM EGTA (Invitrogen) in DMEM:F12 (Invitrogen)]. Resuspension Media [0.05 mg/mL DNase1 (Roche), 10% fetal bovine serum (Wisent Bioproducts) in DMEM:F12 phenol-free medium] was added to each tube and incubated for five minutes. Supernatant was then transferred in Percoll media [19.8% Percoll (GE Healthcare Life Sciences), 2.2% 10xPBS (Wisent Bioproducts) in Resuspension Media], spun down (500 × g, 12.5 minutes, 4 °C), and dissolved in DMEM:F12 phenol-free media. Isolated cells were sorted with a Beckman MoFlo AstriosEQ (Beckman Coulter Canada, Mississauga, ON, Canada) for GFP (for Nestin GFP mice) or YFP (for nDKO and control mice) using the University of Ottawa FACS Core Facility. mRNA was extracted using Arcturus Picopure RNA Isolation Kit (Applied Biosystems, ThermoFisher). For Nestin-GFP samples, RT-PCR was completed using 2.5 ng mRNA and the OneStep RT-PCR kit (Qiagen, Inc.). For nDKO and control samples, RT-PCR was completed using QuantiTect® SYBR® Green RT-PCR Kit in Qiagen Rotor-Gene Q MDx (Qiagen Inc.) and 0.5 ng of mRNA. Primers including PS1-P5:GGCAGCTGAGGCGGAAACCTAGG and PS1-P6:GGATGGCGCTGCTGGAGTGG to target exon 2 and exon 3 of Psen1 (233 bp product)^3^; PS2-P1: TCATGCTATTCGTGCCTGTC and PS2-P2: TACCACGAGGAAGATGGTCA to target exon 4 and exon 5 of Psen2 gene (209 bp product)^7,8^; mGAPDH-P1 and mGAPDH-P2 to target the mouse glyceraldehyde 3-phosphate dehydrogenase gene (571 bp product)^49^.

### Immunohistochemistry and confocal analysis

Coronal brain sections (30 μm) were stained using free-floating procedures as previously described^23^. Briefly, sections were first washed in 1xPBS followed by a blocking incubation step (3% Normal Donkey Serum, 0.1% Tween-20, 0.1% Triton X-100) for 45 minutes. Sections were then incubated in 0.1% Tween-20 and 0.1% Triton X-100 in 1xPBS with primary antibodies, overnight at 4 °C. The primary antibodies used include: chicken anti-GFP (AVES, 1:5000), rabbit anti-DsRed (Clontech, 1:5000), mouse anti-NeuN (Millipore, 1:500), goat anti-DCX (Santa Cruz, 1:500), goat anti-Sox2 (Santa Cruz, 1:500), goat anti-Nestin (R&D, 1:500), mouse anti-GFAP (Millipore 1:500), rabbit anti-Ki67 (cell marque, 1:250). Staining was visualized using secondary antibodies conjugated to CY2, CY3 and CY5 (1:500; Jackson Immunoresearch). Sections were incubated with DAPI (Roche, 1:10000) for ten minutes followed by washing in 1xPBS and mounting.

For quantification of total YFP+ counts, total Ki67+ and DCX+ and counts of GFPCre+ RFP+ and RFP+ cells of virus injected mice, every ninth coronal sections throughout the SGZ were counted by an observer blind to the experimental groups at 400X magnification on the Olympus BX51 fluorescent microscope. For analysis of double- and triple-labeled GFPCre+ cells, all images were acquired on the Zeiss LSM 510 META confocal microscope using multi-track, sequential scanning configuration. Z-series stacks of confocal images were analyzed and rendered in three-dimensions (3D) using the ZEN 2009 software (Zeiss). For analysis of dendritic structure of GFPCre+ RFP+ double positive neurons, 3D projection images were semi-automatically traced with ImageJ software using the NeuronJ plugin. A minimum of 20 cells from each genotype was traced. Sholl analysis was performed using the Sholl analysis ImageJ plugin from the Ghosh lab (http://labs.biology.ucsd.edu/ghosh/software/). Briefly, the analysis was performed by counting the number of times a series of concentric circles (at 5 μm intervals) centered at the cell soma crossed the dendrites of individual cells. A minimum of five individual double transduced (GFPCre+ RFP+) cells per animal, from four animals of each genotype, were analyzed.

### Electrophysiology

Whole-cell electrophysiology was performed as previously described^50,51^. Briefly, adult mice were deeply anesthetized with isofluorane (Baxter Corporation), and transcardially perfused with ice-cold, oxygenated choline-based artificial cerebrospinal fluid (choline-aCSF), containing the following: 119 Choline-Cl, 2.5 KCl, 4.3 MgSO_4_, 1.0 NaH_2_PO_4_, 1.0 CaCl_2_, 11 glucose, and 26.2 NaHCO_3_ (pH 7.2–4). Mice were then decapitated and the brain was quickly removed. Coronal slices (300 μm) containing the full extent of the DG were generated using a vibratome (Leica VT1000S). Brain sections were then transferred to an incubation chamber filled with oxygenated artificial cerebrospinal fluid (aCSF), containing the following: 119 NaCl, 2.5 KCl, 1.3 MgSO_4_, 1.0 NaH_2_PO_4_, 2.5 CaCl_2_, 11 glucose, and 26.2 NaHCO_3_ (pH = 7.2–4). Slices were initially maintained at 30 °C, then allowed to recover at room temperature for at least one hour.

Slices were transferred to a recording chamber and perfused with oxygenated aCSF (2 mL/minute) at room temperature. A Zeiss Axio Examiner Z1 microscope was used to visually target GFPCre+ cells. Borosilicate recording pipettes (4–8 MΩ, World Precision Instruments) were filled with either a cesium- or potassium-based intracellular solutions for voltage- and current-clamp experiments, respectively. The cesium internal solution contained the following (in mM): 115 Cs-methanesulfonate, 0.4 EGTA, 5 tetraethylammonium-Cl, 2.8 NaCl, 20 HEPES, 3 Mg-ATP, 0.5 Tris-GTP sodium salt hydrate, 10 Na-phosphocreatine, and 5 QX-314 (Abcam), (pH 7.2–7.3, 280–290 mOsm/L). The potassium internal solution contained the following (in mM): 115 K-gluconate, 20 KCl, 10 HEPES, 4 Mg-ATP, 0.5 Tris-GTP sodium salt hydrate and 10 Na-phosphocreatine (pH 7.2–7.3, 280–290 mOsm/L). Voltages were uncompensated and liquid junction potentials were left uncorrected. All whole-cell recordings were acquired at 2 kHz (sampled at 10 kHz) using an Axon Multiclamp 700B amplifier and Axon Digidata 1440 A digitizer (Molecular Devices). Synaptic currents were elicited by positioning a borosilicate stimulating pipette (3–5 MΩ, World Precision Instruments) into the middle third of the dentate gyrus molecular layer and electrical stimulation were triggered using an Iso-Flex stimulus isolator controlled by a Master-8 pulse generator (both products from A.M.P.I). These experiments were conducted in the presence of bicuculline methiodide (20 μm; Tocris Bioscience).

For a subset of cells, two-photon imaging was performed to visualize cell morphology. Imaging was conducted using a Ti:Sapphire pulsed laser tuned to 850 nm (MaiTai-DeepSee, Spectra Physics) coupled to a Zeiss LSM710 multiphoton microscope with a 20x (1.0 NA) objective. All electrophysiological recordings were analyzed using Clampfit (Molecular Devices) and OriginPro 8.5 (OriginLab). Results were processed for statistical analysis using Excel (Microsoft), and OriginPro 8.5 statistical software. Passive electrophysiological properties reported by the membrane test of Axon Clampex (Molecular Devices) were acquired immediately after whole-cell break-in. Time constant was calculated from fitting a single exponential function from a subthreshold current pulse (−20 or +20 pA, 1 s) in current clamp. Action potential properties were measured using the threshold search function of Clampfit (Molecular Devices). AMPA:NMDA ratio was calculated at +40 mV as previously described^50,52^. Briefly, the AMPA current value was estimated at +40 mV, at the duration of expected peak current of the AMPA response evaluated at −70 mV, whereas the NMDA current value was obtained at +40 mV, at 3× decay time constant of the AMPA current at −70 mV.

### Statistics

All data are reported as mean ± S.E.M. and the statistical analysis was performed using GraphPad Prism (v6.0) software. Table 1 provides a summary of statistical test used and outcome obtained. Experiments with two groups were analyzed by the two-tailed *student’s t-test*. Analyses of three or more groups were performed using an ANOVA test followed by a Tukey’s *post hoc*. Statistical significance was defined as *p* < 0.05.

**Table 1:** Summary of Statistical Analysis and Outcomes.

Figure	Distribution	Type of Test	P Value
1B	Normal	Two-tailed t-test	0.5723
1D	Normal	Two-tailed t-test	0.1467
1G	Normal	Two-tailed t-test	0.6392
1H	Normal	Two-tailed t-test	0.7904
2C	Normal	Two-way ANOVA	0.0007
2D	Normal	Two-way ANOVA	0.7136
3B	Normal	Two-tailed t-test	0.8664
3D	Normal	Two-tailed t-test	0.4834
3F	Normal	Two-way Repeated measure ANOVA	0.9659
4B	Normal	Two-tailed t-test	R_M_: 0.92166 V_Rest_: 0.08921 C_M_: 0.5814
4D	Normal	Two-tailed t-test between Control and vDKO	First 2 AP (Amp.): 0.4314 Last 2 AP (Amp.): 0.58094 First 2 AP (Time): 0.36579 Last 2 AP (Time): 0.20101
4F	Normal	Two-tailed t-test	0.5257
5C	Normal	Two-way ANOVA	<0.0001
5D	Normal	Two-way ANOVA	0.6693
6C	Normal	Two-tailed t-test	0.0030
6E	Normal	Two-way ANOVA	0.0018
7B	Normal	Two-tailed t-test	0.5437
7D	Normal	Two-tailed t-test	0.8526
7F	Normal	Two-tailed t-test	0.3641
7H	Normal	Two-tailed t-test	0.7040

## Electronic supplementary material

Supplemental Information
