# Supplementary material for: Adult hippocampal neurogenesis occurs in the absence of Presenilin 1 and Presenilin 2
Source: Sci Rep. 2018 Dec 18;8:17931. doi: 10.1038/s41598-018-36363-7 (PMC6299003; doi:10.1038/s41598-018-36363-7)
Supplement: Supplementary file 1 — Supplemental Information [file 41598_2018_36363_MOESM1_ESM.pdf]

Supplemental Documents

Adult hippocampal neurogenesis occurs in the absence of *Presenilin 1* and *Presenilin 2*

Jagroop Dhaliwal[1], Timal S. Kannangara[1], Michael Vaculik[1], Yingben Xue[1], Keren L. Kumar[1], Amanda Maione[1], Jean-Claude Béïque[1], Jie Shen[2] and Diane C. Lagace[1]*

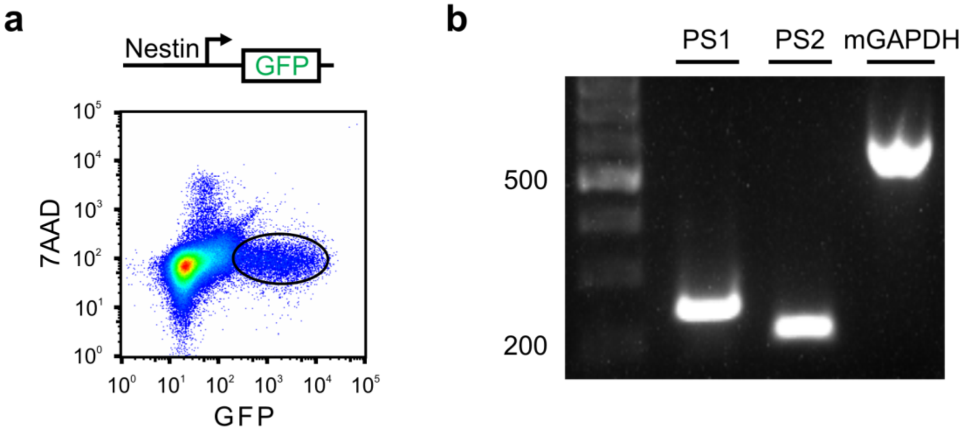

Supplemental Figure 1. Presenilins are expressed in Nestin-GFP-expressing stem and precursor cells in the dentate gyrus. a) Density-scatter plots of FACS-isolated cells from the dentate gyrus of adult Nestin-GFP mice. **b**) Sorted GFP+ cells express mRNA for PS1, PS2 and control mGAPDH.

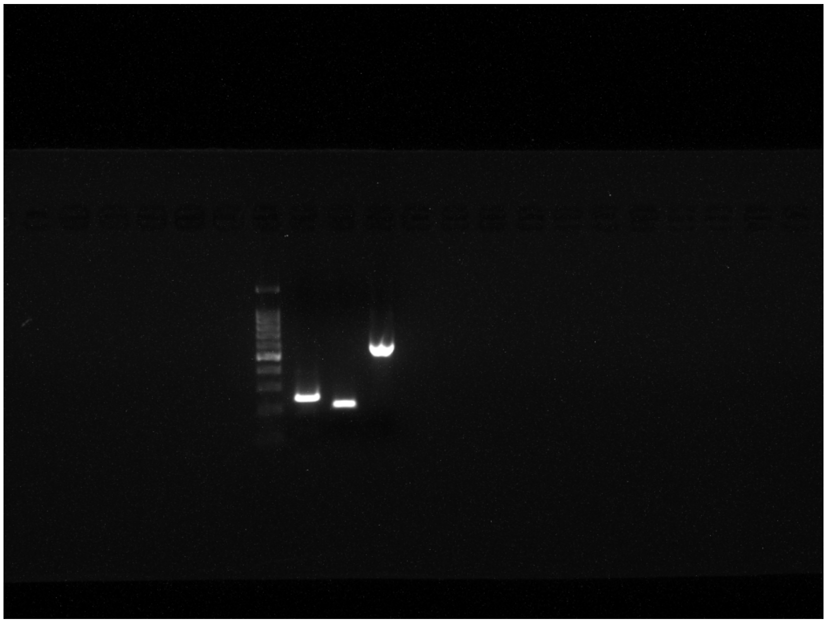

Supplemental Figure 2. Full-length Gel presented in Supplemental Figure 1.

	WT	Control	vDKO
**Membrane Resistance (R_M; MΩ)**	341.3 ± 78.0	417.4 ± 54.0	426.8 ± 71.6
**Resting Membrane Potential (V_{Rest}; mV)**	-81.02 ± 2.1	-82.5 ± 2.4	-75.5 ± 2.8
**Estimated Cell Capacitance (C_M; pF)**	45.8 ± 8.0	48.8 ± 3.5	61.7 ± 19.7
**Estimated Tau (ms)**	33.2 ± 3.7	46.4 ± 6.9	37.1 ± 5.7
**Action Potential Amplitude (First Two Spikes) (mV)**	109.5 ± 5.6	117.2 ± 1.6	113.7 ± 3.5
**Time to Peak (First Two Spikes) (ms)**	1.92 ± 0.03	1.62 ± 0.03	1.70 ± 0.07
**Action Potential Amplitude (Last Two Spikes) (mV)**	107.3 ± 5.0	103.3 ± 2.5	100.2 ± 4.4
**Time to Peak (Last Two Spikes) (ms)**	2.04 ± 0.05	1.86 ± 0.05	2.03 ± 0.11
**N (cells/mice)**	5/2	7/3	9/3

Supplemental Table 1. Electrophysiological properties of GFPCre+ virally-labeled NPCs. Related to Figure 4. Data are presented as the mean ±SEM.
